# Cancer Evolution and the Limits of Predictability in Precision Cancer Medicine

**DOI:** 10.1016/j.trecan.2015.11.003

**Published:** 2016-01

**Authors:** Kamil A. Lipinski, Louise J. Barber, Matthew N. Davies, Matthew Ashenden, Andrea Sottoriva, Marco Gerlinger

**Affiliations:** 1 Centre for Evolution and Cancer, The Institute of Cancer Research, London, UK; 2 Gastrointestinal Cancer Unit, The Royal Marsden Hospital, London, UK

**Keywords:** precision cancer medicine, cancer genetics, cancer evolution, predictive biomarkers, prognostic biomarkers, drug resistance

## Abstract

The ability to predict the future behavior of an individual cancer is crucial for precision cancer medicine. The discovery of extensive intratumor heterogeneity and ongoing clonal adaptation in human tumors substantiated the notion of cancer as an evolutionary process. Random events are inherent in evolution and tumor spatial structures hinder the efficacy of selection, which is the only deterministic evolutionary force. This review outlines how the interaction of these stochastic and deterministic processes, which have been extensively studied in evolutionary biology, limits cancer predictability and develops evolutionary strategies to improve predictions. Understanding and advancing the cancer predictability horizon is crucial to improve precision medicine outcomes.

## Trends

Large-scale genomic cancer medicine initiatives are under way in several countries across the globe.

However, it remains a major challenge to use genomic information to make accurate predictions for individual cancer patients.

Multiple genomic studies substantiated the notion of cancer as an evolutionary process that can readily adapt within the lifetime of a patient.

Evolutionary adaptation results from the interplay of mutation generation and genetic drift, which are both stochastic processes, and clonal selection, which is deterministic in nature.

The influence of stochastic factors fundamentally limits the predictability of cancer evolution.

Understanding the limits of predictability and the development of more accurate prediction algorithms using evolutionary models is key to improving outcomes through genomic cancer medicine.

## Cancer as an Evolutionary Process

The ability to precisely predict the future clinical course of an individual patient's cancer would be highly beneficial for oncological care. For example, patients whose cancers will never progress to the point of affecting their health may not require any treatment and those who need systemic therapy should only be treated with drugs that have a realistic chance of being effective.

Genomic aberrations differ between cancers of the same histological type, to the extent that no two tumors are thought to show an identical somatic genetic aberration profile [1]. The specific combination of somatic genetic and epigenetic aberrations within a tumor, in the context of the germline variants present in the same patient, is thought to be a major determinant of the biology and hence of the clinical course of a cancer. Recognition of this intertumor heterogeneity led to the concept of personalized cancer medicine: deciphering individual cancer genomic profiles should provide precise insights into disease biology and allow the targeting of genetically encoded susceptibilities for therapeutic benefit. Next-generation sequencing technologies enable the routine interrogation of these (epi)genomic landscapes 2, 3. In parallel, an increasing number of cancer drugs expand the therapeutic options to target specific genetic alterations. Yet, despite noticeable advances of personalized therapy approaches in some tumor types, the ability to predict whether and for how long an individual cancer will respond to therapy and what genotype will eventually evolve to drive resistance remain suboptimal [4]. Precisely forecasting whether a cancer will recur after potentially curative therapy remains even more elusive, resulting in dramatic overtreatment in oncology [5].

Forty years ago, Peter Nowell first formally described cancer as an evolutionary process [6]. This hypothesis has since been substantiated by the discovery of intratumor subclonal heterogeneity and ongoing clonal selection in multiple cancer types 7, 8, 9, 10, 11, 12, 13. Recognition of this fundamental evolutionary nature of cancer and the notion of tumors as dynamically adapting ‘organisms’ requires a reassessment of the opportunities and limitations this bestows on precision cancer medicine.

‘Nothing in biology makes sense except in the light of evolution’ – Theodosius Dobzhansky

**Figure 1:**
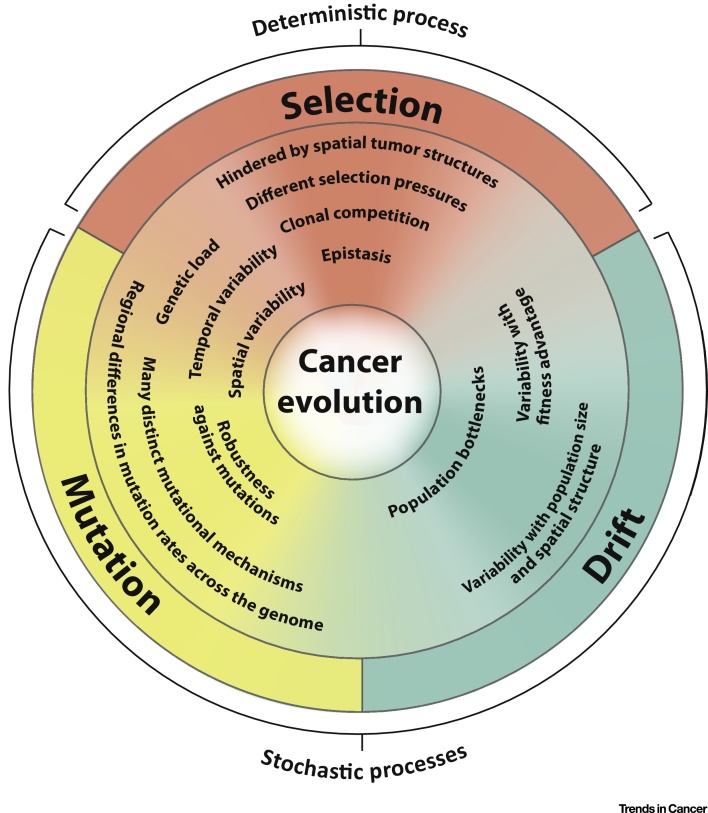
Key Figure: Mutation, Selection, and Drift are the Three Basic Processes Shaping Cancer Evolution

Cancer evolution is conceptually similar to the evolution of asexual microorganisms [14] and should be governed by the dynamic interplay of the same three basic processes [15]: (i) the generation of heritable variation; (ii) the influence of random birth and death events on the fate of new genotypes, referred to as genetic drift; and (iii) Darwinian selection, which changes the frequency of genotypes in the population based on their relative fitness advantage (Figure 1, Key Figure).

The acquisition of heritable alterations and genetic drift are both random processes, while Darwinian selection is deterministic in nature (**deterministic process**; see Glossary) 16, 17. This questions to what extent cancer evolution and hence the future clinical course of a patient can be predicted with precision. This review integrates results from recent cancer genomics studies with fundamental evolutionary biology concepts to assess how stochasticity (**stochastic process**) and spatial structures limit cancer predictability. Based on this evolutionary perspective of cancer, we subsequently assemble novel approaches such as genetic micro- and macroheterogeneity profiling and the application of empirical cancer fitness landscapes, which should expand the predictability horizon for precision cancer medicine efforts.

## Mutation Generation

Heritable somatic variation encompasses genetic alterations such as point mutations, insertions, deletions, and chromosomal aberrations, as well as random epigenetic changes that are heritable over cell generations. For simplicity, the term ‘mutation’ is used for all heritable somatic alterations throughout this review.

A baseline mutation rate can be detected in any mitotic tissue, but mutation rates are often elevated in cancer [18]. Mutations can result from cell extrinsic (e.g., tobacco smoke exposure) or intrinsic processes (e.g., oxidative damage or defects in DNA repair). Many mutational processes preferentially strike in specific DNA sequence contexts, biasing mutations towards genomic regions in which these are overrepresented. Distinct mechanisms can hence leave specific footprints or mutational signatures in the genome, as shown by a pan-cancer analysis that revealed 20 different mutational signatures, nine of which could be linked to known molecular mutational mechanisms [19]. The preferential deamination of cytosine in 5′-TC-3′ dinucleotides and regional hypermutation clusters caused by the aberrant activity of the apolipoprotein B mRNA editing enzyme catalytic polypeptide-like (APOBEC) RNA-editing enzymes is one example [20]. Late-replicating genomic regions are more prone to acquire mutations than early replicating regions [21], and chromatin organization further influences regional mutation rates [22], contributing to variable mutation rates in different genomic regions.

Structural aberrations also result from diverse molecular mechanisms. Fusion of two chromosome ends fostering cycles of chromosome breakage and fusion during mitosis [23] or catastrophic ‘chromothripsis’ events leading to massive genomic rearrangements within a single cell division [24] are two examples. DNA fragments can even be detached from chromosomal DNA and propagated as so-called ‘double minute chromosomes’ whose abundance can change rapidly, for example, to maintain optimal epidermal growth factor receptor (*EGFR*) signaling levels during cancer drug therapy [25].

Different mutational processes can predominate at different times. Clear cell renal cell carcinomas (ccRCC) and non-small cell lung cancers (NSCLCs) both exhibited distinct mutational signatures during early carcinogenesis compared with cancer progression and between different tumor subclones 9, 26. Ongoing tobacco exposure had a minor influence on mutation generation during NSCLC progression where mutations were predominantly induced by APOBEC enzymes [26]. Single cell sequencing of two breast cancers showed that point mutations were generated continuously during cancer progression, whereas copy number aberrations had been acquired early [27]. Whole genome doubling events can lead to tetraploidy, which is permissive for further chromosome gains and losses [28]. Genome doubling can occur early in carcinogenesis [26] but also late during cancer progression [9]. Extra gene copies acquired through genome doubling may buffer potentially deleterious effects of new mutations [9]. Genome doubling might therefore not only catalyze mutation generation but also increase mutation tolerance.

Cancer originates from a single cell with a diploid genome. This encodes the blueprint for embryological development and adult homoeostasis of a complex multicellular organism and is also structurally optimized to undergo meiosis and recombination during sexual reproduction [15]. Such constraints on genome structure and many genes regulating tissue-specific functions are likely to be irrelevant for cancer cells, which permits their survival despite highly aberrant genomes. This mutational robustness allows cancers to probe a vast genomic space for novel phenotypes 29, 30.

Taken together, mutations are the prerequisite for cancer evolution. Mutation rates, the genomic regions that are prone to mutagenesis, and the timing when particular mutagenic processes operate during cancer progression can vary significantly between but also within individual cancers. This influences the accessibility of novel genotypes and phenotypes and hence the opportunities for evolution, as shown for APOBEC-driven mutagenesis, which generates activating phosphoinositide (PI)3-kinase mutations in many cancers where it is active [31]. Yet, even if the mutational mechanisms operating in a cancer cell could be measured exactly, mutations still occur randomly with regard to their timing and exact genomic location.

## Drift

Genetic drift refers to changes of the frequency of an allele in a population due to random birth and death events: each cell in a newly generated cancer subclone has a certain probability of dying as a result of random factors and occasionally all cells of a small subclone die, even if this clone harbors a highly beneficial mutation. Drift has a bigger impact in smaller populations [15] and is more likely to eradicate a single cell or a small clone that has not yet expanded significantly. Drift is more pronounced after population bottlenecks, for example, when a few or single cells colonize a new metastatic niche or after a massive reduction in population size through cytotoxic treatment. As a consequence of drift, the expansion of a clone with a beneficial mutation may not be predictable with certainty until this clone exceeds a certain abundance at which it escapes potential extinction through drift [32].

Drift influences cancer initiation 33, 34 but experimental data demonstrating the strength of this effect in cancer progression is lacking. New technologies assessing clonal composition at the single cell level [27] or clonal dynamics through lineage tracing in model systems 33, 35 may provide such insights.

## Selection

A new mutation that increases the ability of the cell to survive and reproduce under particular environmental conditions and that has escaped drift will gradually increase in its abundance within the population. This clonal selection is arguably the only deterministic force in evolution 16, 36.

Next-generation sequencing technologies revealed these clonal selection processes for the first time in detail and drafted the first chapters of cancer evolution rulebooks. Multiple intratumoral subclones harboring different driver mutations, displaying distinct phenotypes, and evolving with branched phylogenies were identified in many cancer types 7, 9, 11, 13, 37, 38, 39, 40, 41.

The presence of multiple subclones within a tumor can lead to clonal competition. The fitness of an individual subclone is then defined in relation to the fitness of other competing clones [42]. Hence, beneficial mutations that escape the potentially deleterious effects of drift can still be eradicated by competing clones, complicating the prediction of evolutionary outcomes.

The identification of spatially separated subclones in many solid tumors suggests that their 3D structure hinders intermixing of subclones 9, 11, 26, 40, 43. Such spatial constraints most likely limit clonal competition to the immediately neighboring subclones and even highly fit subclones may never be able to rise to 100% abundance, an event referred to as ‘fixation’ or ‘selective sweep’ in evolutionary biology. Solid tumor spatial structures may therefore augment the generation and maintenance of subclonal heterogeneity and drive the system towards a more stochastic behavior. This notion is supported by microbial experiments that found higher intrapopulation genetic heterogeneity in spatially structured environments [44]. Thus, solid tumors may be ecological microcosms composed of myriads of small and localized populations, each competing only at its edges with neighboring populations.

## The Selection of Drug Resistant Clones

Resistance almost invariably develops during drug therapy in metastatic tumors and studies into the origins of acquired resistance impressively illustrated the evolutionary plasticity of cancer.

For example, the majority of NSCLCs treated with first generation EGFR inhibitors such as gefitinib or erlotinib acquire resistance through the evolution of *EGFR* T790M mutations [45]. Alternative *EGFR* mutations, MET proto-oncogene or erb-b2 receptor tyrosine kinase 2 (*ERBB2)* amplification or non-pathway-dependent resistance through transformation into small-cell lung cancers were observed less frequently in biopsies from resistant tumors [46]. The high prevalence of T790M-driven resistance led to the development of third generation EGFR inhibitors such as rociletinib, which are active against this oncoprotein and achieved response rates of 59% in T790M NSCLCs [47]. Rebiopsies after rociletinib failure found that 6/13 resistant tumors were T790 wild-type (wt) again. These resistant clones were already present before rociletinib therapy initiation and probably harbored alternative resistance drivers to first generation inhibitors [48]. Thus, subclonal heterogeneity was a key driver of treatment failure. C797S *EGFR* mutations are an alternative resistance mechanism to third generation EGFR inhibitors [49]. Importantly, EGFR signaling could still be inhibited with a combination of first and third generation inhibitors if the C797S mutation was located in trans with T790M but this combination was ineffective if these were located in cis on the same *EGFR* allele. As C797S mutations occur randomly on one of the two *EGFR* alleles, the optimal further therapy cannot be predicted until the mutational event has occurred and has been detected. This compellingly demonstrates how stochastic events can limit predictability.

Somatic mutation detection in circulating tumor DNA (ctDNA) is likely to provide a more comprehensive overview over the subclonal heterogeneity of solid tumors than single biopsies. ctDNA analysis indeed detected up to 12 distinct subclones, each harboring a different mutation in RAS-type family GTPases (*RAS*) or v-Raf murine sarcoma viral oncogene homolog B1 (*BRAF*) genes, in individual patients with colorectal cancer (CRC) after they had developed anti-EGFR therapy resistance [12]. Polyclonal resistance has also been identified in other tumor types after the failure of targeted drugs, hormones, or chemotherapy 13, 50, 51, 52, 53. Polyclonal resistance may thus be a common phenomenon in solid tumors, demonstrating the enormous evolutionary adaptability of cancer. Clonal dynamics analyses in the ctDNA from CRC patients further suggested that Kirsten rat sarcoma viral oncogene homolog (*KRAS*) resistance mutations had been present in small subclones before anti-EGFR therapy initiation [8]. Thus, the standing genetic variation in cancers has been recurrently found to provide a reservoir of phenotypes permitting evolutionary rescue from extinction in changing environments.

Overexpression of the BRAF V600E oncoprotein caused resistance but also dependency on BRAF inhibitor therapy in melanoma xenografts [54]. Whether such drug resistant cells were present before BRAF inhibitor therapy is unclear but it is conceivable that their fitness disadvantage would drive them to extinction in the absence of selection pressure. This illustrates how fitness differences in the presence or absence of drug may influence the probability of pre-existence of specific resistance drivers. Negative fitness effects may also explain the decline of drug resistant CRC subclones after withdrawal of anti-EGFR therapy [55].

Taken together, pervasive drug resistance evolution demonstrates that neither the acquisition of resistance driver mutations nor the potential elimination by drift are limiting factors for evolutionary adaptation in these tumor types. Despite the occurrence of therapeutically challenging polyclonal resistance, only a small fraction of possible resistance genotypes appears to be frequently accessed, implying a degree of evolutionary predictability. This questions which cancer characteristics favor the predictability of resistance genotypes, which may permit more effective pre-emptive interventions.

## Cancer from a Population Genetics Perspective

Studies in microbes extensively investigated how key population genetics parameters – mutation rate, population size, and the strength of selection – alter predictability and chance in evolution [56]. Understanding how the same three parameters influence cancer evolution is crucial to further outline the limits of cancer evolution predictability.

‘Nothing in evolution makes sense except in the light of population genetics’ – Michael Lynch [15]

### The Impact of Mutation Rate and Population Size

The supply of new mutations is a limiting factor for adaptation in small cancers with no or minimal genomic instability. As the generation of advantageous mutations and escape from drift are stochastic events, the time to the emergence of a new characteristic and its exact genotype cannot be predicted accurately. It is unlikely that multiple clones with increased fitness are present at the same time in such a cancer. A new beneficial mutation that has sufficiently expanded to escape drift is therefore likely to deterministically steer the evolutionary track of the population. Once this subclone becomes detectable in a patient, the further clonal expansion process may be highly predictable.

Chronic myeloid leukemia (CML) in the chronic phase is a cancer type in which the mutation supply is usually limited. It is genetically stable and has a small effective population size as it is maintained by a small pool of cancer stem cells [57]. Only mutations generated in a cancer stem cell can be of relevance for evolution; all others will invariably go extinct as a consequence of the limited replicative potential of non-stem cells [58]. The time from treatment initiation with imatinib, an inhibitor of breakpoint cluster region and ABL proto-oncogene 1 (*BCR-ABL*) fusion protein, until a resistance mutation becomes detectable varies highly between patients, probably as a consequence of the stochasticity of mutation generation and drift. But once such a mutation is detectable, most cancers will progress, suggesting that these clones entered a deterministic and predictable trajectory. Distinct resistance mutations in the *BCR-ABL* gene have different fitness effects in the presence of imatinib and the time from detection of a resistance mutation to progression can be estimated with higher precision when the exact mutation is taken into account 59, 60.

**Figure 2:**
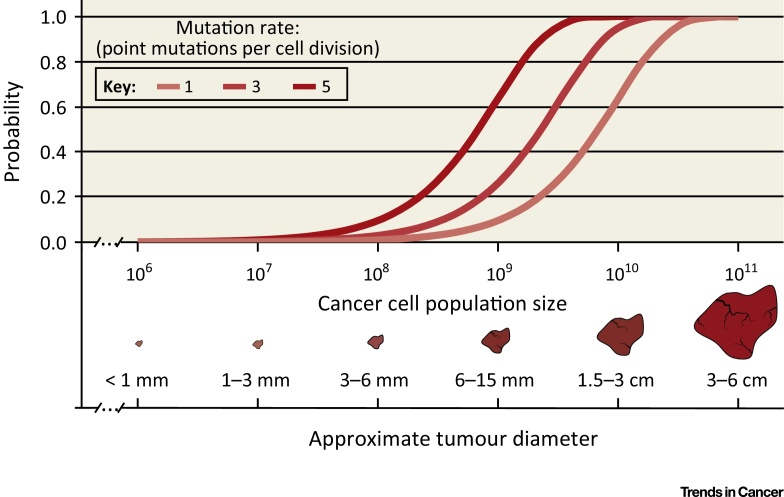
Probability of Occurrence of a Specific Point Mutation in the Cancer Genome. The probability that a specific point mutation occurs at least once during the growth of a tumor to the indicated population size is shown. Calculations were performed for three different mutation rates covering mutation rate ranges observed in non-hypermutator human cancers [27]. The probability for such a mutation converges to 100% for cancer sizes that are typical for patients requiring systemic therapy, regardless of the mutation rate. Thus, any specific resistance driver mutation has most likely been generated at least once in an advanced solid tumor. For simplicity, this model only assesses the probability of mutation generation and does not take into account that these can be eradicated by drift. Absence of cell death and constant mutation rates across the genome and for all possible single base substitutions were simplifying assumptions. The probability was calculated as 1 – (1 – *k*)*^N^*. *N* is the number of total cell divisions (which is equal to population size – 1). *k* is the probability of occurrence of a specific mutation during a cell division, calculated as *m/G* × 1/3, where *m* is the mutation rate per cell division and *G* is the size of the haploid human genome (3.3 × 10^9^ bp). Approximate tumor diameters were calculated based on [107].

All else being equal, the supply of new mutations increases with the cancer cell population size and the probability that a specific advantageous mutation will occur converges towards 100% in advanced cancers, which can harbor hundreds of billions of malignant cells (Figure 2). The likelihood of resistance development should hence increase and time to resistance decrease with the cancer population size, which is supported by clinical observations 61, 62. Large population sizes further favor the simultaneous emergence of multiple beneficial mutations in different cancer cells, leading, for example, to polyclonal resistance. Yet, the increasing spatial segregation of tumor subpopulations is likely to hinder competition and to foster genetic heterogeneity as outlined. Segregation into many small populations may further increase the influence of stochastic drift. Cancer cell motility [63] or reseeding between metastases 10, 13, 64 may mitigate the impact of spatial tumor structures, but further studies are necessary to assess the prevalence of these processes.

### The Role of Detrimental Mutations

**Figure 3:**
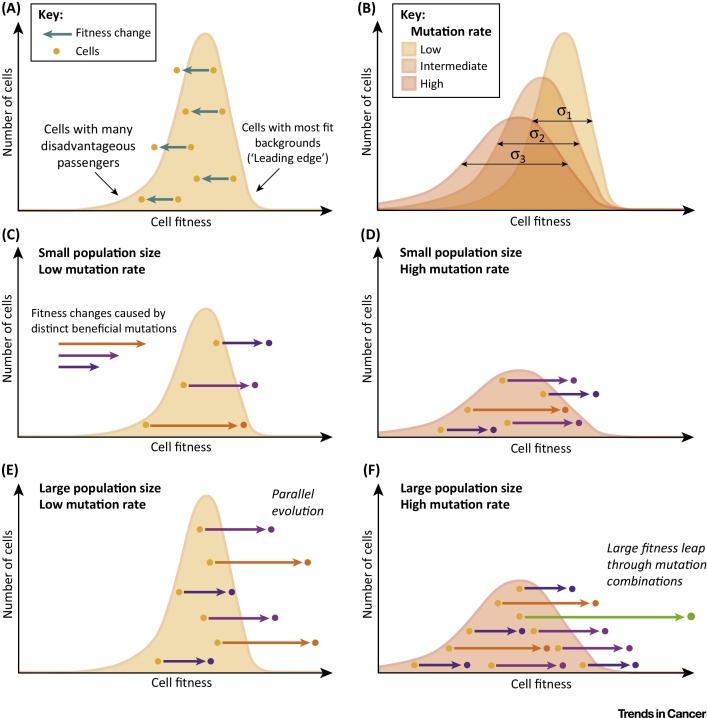
Fitness Effects of New Mutations Arising in Cancers with Different Population Sizes and Mutation Rates. (A) The accumulation of disadvantageous mutations causes negative fitness shifts in affected cells and their progeny. Stepwise acquisition of multiple disadvantageous mutations pushes cells towards the left side of the graph. The fitness distribution can be approximated by a Poisson distribution in large populations with a relatively low mutation rate [66]. (B) A high mutation rate leads to a large genetic load that increases the variance of the fitness distribution and decreases overall population fitness [66]. (C) The impact of a new beneficial mutation depends on the initial fitness of the cell in which it occurs. A slightly advantageous mutation (purple) can be more likely to be successful in evolution than a mutation with a large beneficial effect (pink) if it occurs in a fitter cell. (D) High mutation rates increase the supply of beneficial mutations but also cell-to-cell variation in fitness, thus hindering selection of the most advantageous mutations. (E) In large populations the same mutation is likely to arise independently in different cells, leading to parallel evolution. These clones may still differ in overall fitness owing to variable loads of disadvantageous mutations. (F) A rare combination of mutations can produce large and unpredictable fitness leaps. This most likely occurs in large and genetically unstable tumors.

Genomic instability can increase the supply of beneficial mutations in small cancers to levels equal to or exceeding those of large but genomically stable tumors. A distinct difference is that multiple mutations are likely to arise and accumulate within individual cells in the former scenario. Thus, additive and epistatic effects increasingly influence overall subclonal fitness in genomically unstable tumors. Mutations can impair fitness through multiple mechanisms including loss-of-function, detrimental neo-functions, cellular stress induced by misfolded or aberrantly expressed proteins, fatal structural aberrations in the genome, or by increasing cancer immunogenicity [65]. Thus, it is likely that many mutations have at least mildly disadvantageous effects, despite the high mutational robustness of cancer genomes. Such mutations accumulate in the cancer cell population and the evolutionary success of a new advantageous mutation that randomly originates in a single cancer cell becomes increasingly influenced by the net fitness effect of all somatic mutations within that cell [66] (Figure 3). This has been shown in microbial evolution experiments, where the most successful mutations were often those that were fortunate and occurred in the best genetic backgrounds [67]. Cell-to-cell differences in the mutation load hence diminish the ability to accurately predict the impact of a given driver mutation before it has actually occurred in a random cell of a heterogeneous tumor cell population.

Mathematical models [16] and yeast evolution experiments 32, 67 suggest that determinism becomes further disrupted in very large populations through the rare occurrence of highly fit clones that acquired fortuitous combinations of several mutations. Such mutations may even be disadvantageous individually; success in evolution then requires epistatic cooperation through co-occurrence within the same genome. Catastrophic events such as chromothripsis that generate multiple genetic aberrations [20] or chromosomal instability, which alters the gene dosage of multiple genes colocalizing on a DNA segment [68], are possible one-step mechanisms to acquire beneficial driver combinations. The limited clonal competition in solid tumors coupled with the mutational robustness of cancer genomes may permit the survival of large numbers of subclones with high mutation loads that occasionally facilitate such unpredictable trajectories.

The irreversible accumulation of detrimental mutations in asexually reproducing organisms may eventually lead to their extinction, a phenomenon referred to as Muller's ratchet in evolutionary biology. Sequencing of ultra-hypermutator cancers in children with germline mutations in the proofreading DNA polymerase epsilon (*POLE*) indeed found a maximum of ∼20 000 exonic mutations [30]. This may indicate a ‘mutational ceiling’ beyond which the detrimental effects of such large mutation loads cannot be tolerated. Despite this, the tumors had not regressed spontaneously and were removed surgically. Moreover, mutation loads are magnitudes lower in most other hypermutator cancers [18] and the rarity of spontaneous cancer regression events questions the relevance of extinction driven by high mutational loads. Nevertheless, the prognosis of tumors with hypermutator phenotypes [69] or high levels of chromosomal instability [70] can be better than in those with intermediate genomic instability levels, suggesting that negative fitness effects of large mutational loads may be relevant for precision cancer medicine.

**Figure 4:**
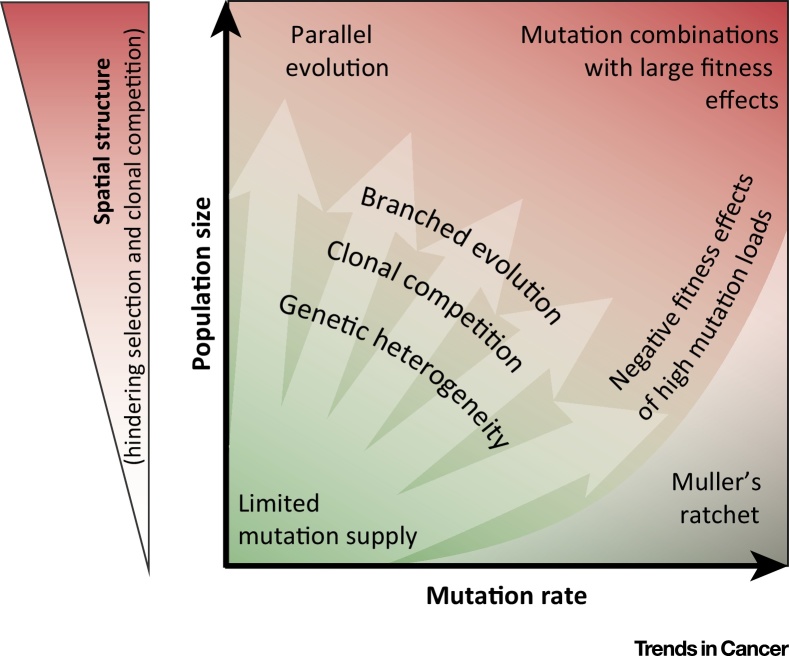
Cancer Evolution Features by Population Size and Mutation Rate. Evolvability is low in cancers where the mutation supply is a limiting factor but increases with population size and/or mutation rate (large arrows). The features of cancer evolution that are thought to predominate at specific combinations of mutation rate and population size are indicated in the figure. Spatial structures increase and clonal competition decrease with the population size and further impact evolutionary outcomes as described in the text.

Overall, the relationships between population size, mutation rate, and predictability are complex (Figure 4) and non-monotonic [16]. Time-to-event predictability may be particularly difficult in tumors with a limited mutation supply due to the influence of stochastic mutation generation and drift events. Predictability may be higher in intermediate size tumors with low instability where the supply of advantageous mutations is relatively large and may decline in large tumors with spatial structures and in those with high instability where unexpected mutation combinations arise. It is conceivable that therapeutic strategies that aim to minimize the size of the cancer cell population throughout the patient history may restrain evolvability and polyclonal resistance development. Reducing mutation rates, for example, by inhibiting enzymes such as APOBEC that drive genomic instability processes [71] could be further tractable approaches to control evolvability. Mathematical models further suggested that rationally selected and administered combination therapies could thwart the evolution of drug resistance 72, 73. Yet, this strategy is often limited in practice as a result of the overlapping toxicity profiles of many cancer drugs and can lead to untoward drug interactions [74].

## Impact of the Strength of Selection

A challenge for the quantification of the fitness of cancer subclones is that it depends on the selection pressures operating in an individual cancer. Selection can, for example, vary between tumor types as shown by the detection of star-shaped phylogenies in primary CRCs, which suggested the absence of strong selection [75], whereas ccRCCs showed evidence for ongoing evolution in primary tumors [11]. It is conceivable that tumors in which selection appears absent may be those that already harbor strong driver genes and proliferate rapidly. Thus, the acquisition of additional drivers may have only minimal impact on their growth dynamics and selection may be too weak to shift clonal compositions detectably 76, 77. Yet, selection pressures may change dramatically when cancers increase in size. For example, hypoxia is likely to increase in a growing tumor and can select for tumor protein p53 (*TP53*) mutant clones that evade apoptosis under these hostile conditions [78]. Assessing tumor microenvironmental features, such as blood vessel densities or immune cell infiltrates [79], can reveal some of the selection pressures that are relevant in an individual tumor. Cancer cells colonizing metastatic sites are also likely to encounter altered selective landscapes. This most likely explains why small subclones rather than the dominant clone in the primary tumor seeded multiple metastases in breast, prostate, renal, and pancreatic primary cancers 10, 13, 39, 64, 80, 81 and why genomic landscapes of metastases differed between colonized organs 80, 82. Approaches to predict metastatic progression may need to consider which genotypes are most likely to be viable in candidate metastatic organs, rather than at the primary tumor site. Genetic analyses of tissues from metastases and primary tumors are necessary to reveal recurrently selected genetic alterations that permit colonization of distinct organs.

In summary, cancer evolution is influenced by various selective pressures that can act simultaneously and vary in space and time [83], challenging the simplified perception of evolutionary adaptation as movement on a static fitness landscape 84, 85. Drug therapy may largely be an exception as conventional dose regimens based on maximum tolerated doses probably apply a uniform selection pressure. Predicting drug resistance may therefore be an easier task than predictions of tumor progression and recurrence.

## Expanding the Predictability Horizon

Cancer evolution is a complex and dynamic process governed by simple principles (Figure 1). The spatial structure of solid tumors likely increases the genetic diversity that can be maintained in the cancer cell population, augments the influence of stochastic factors, and reduces the efficacy of selection, which is the only deterministic force in cancer evolution. Yet, there are opportunities to improve cancer precision medicine predictability by applying evolutionary principles.

### Macroheterogeneity Profiling

Once a subclone has expanded and escaped drift, its fate is increasingly determined by its fitness advantage. Methods that can identify such evolving macroscale subclones and estimate their fitness advantage may permit the accurate prediction of short-term dynamics of heterogeneous tumors. For example, detection of pre-existing drug resistant subclones before anti-EGFR therapy in NSCLCs [86] or in CRCs [87] allows more accurate estimates of progression free survival times and the presence and evolution of subclonal driver mutations correlated with worse outcomes in chronic lymphocytic leukemia [88].

Current genetic prediction approaches are largely based on the analysis of recurrent driver aberrations. This overlooks negative fitness effects of so-called passenger mutations, which most likely diminishes predictive accuracy. Efforts to identify non-synonymous mutations that generate neo-epitopes [65], which impair cellular fitness due to resulting immune recognition, are important steps to improve clonal fitness analysis. Further methods to predict the selective advantage or disadvantage of non-recurrent alteration are clearly necessary.

Mathematical models suggest that at the time of treatment, each metastatic lesion may contain several drug resistant subclones that differ in their population sizes by orders of magnitude [89]. Rational targeting of the most abundant resistant clone is likely to foster the outgrowth of smaller clones, which may be driven by distinct and untargeted resistance mechanisms, as shown for T790M NSCLC treated with third generation EGFR inhibitors [48]. These complexities of cancer clonal compositions require tracking systems that update forecasts regularly. This may be achievable through ctDNA analysis technologies that permit increasingly sophisticated subclonal detection and tracking 12, 55, 90, 91. The development of evolutionary forecasting methods that are applicable to such data should be a priority in precision medicine.

Taken together, detecting macroscale clones that are expanding almost deterministically and precise quantification of the fitness effects of somatic mutations within such clones may significantly expand the predictability horizon. Tracking of subclonal composition through ctDNA could also inform pre-emptive therapeutic switch strategies as soon as evolving resistant subclones become detectable. By keeping the cancer cell population size small, such adaptive therapy approaches could help to restrain evolvability and the development of polyclonal resistance.

### Microheterogeneity Profiling

Genetic analyses at the macroscale predominantly reveal mutations present in large subclones that have already been successful in evolution and provide an ‘archaeological’ record of past mutational processes. Yet, this overlooks the mutation generation ongoing at the single cell level and the heterogeneity confined to small subclones of up to a few thousand cancer cells [77]. This unselected standing genetic variation at the microheterogeneity scale arguably confers the majority of the mutational load of a cancer cell population. This may indeed be the most critical determinant of cancer evolvability, akin to the engine of cancer evolution that generates the heritable phenotypic diversity that selection can act upon. Novel approaches to sequence small cancer cell subpopulations, such as CRC crypts 92, 93 or single cells [27], are starting to provide detailed insights into microscale heterogeneity.

The combined measurement of standing genetic variation and mutational processes through microscale sequencing, together with cancer cell population size estimates obtained through routine imaging, may categorize individual tumors into subgroups differing in their overall evolvability and predictability. Quantifying the size and restricting sequencing to the cancer stem cell pool will be critical in cancers maintained by stem cells to avoid population size overestimates and the erroneous interpretation of mutations confined to non-stem cells that are destined for extinction [58]. Single cell sequencing from tumor biopsies that are subject to sampling biases 11, 13, 26 clearly underestimates the overall complexity of many tumors. However, combining microheterogeneity analyses with macroscale assessments of deterministically expanding tumor subclones and their dynamics through ctDNA techniques may mitigate individual disadvantages and allow the most precise forecasting.

## Cancer Evolution as Movements on a Fitness Landscape

**Figure 5:**
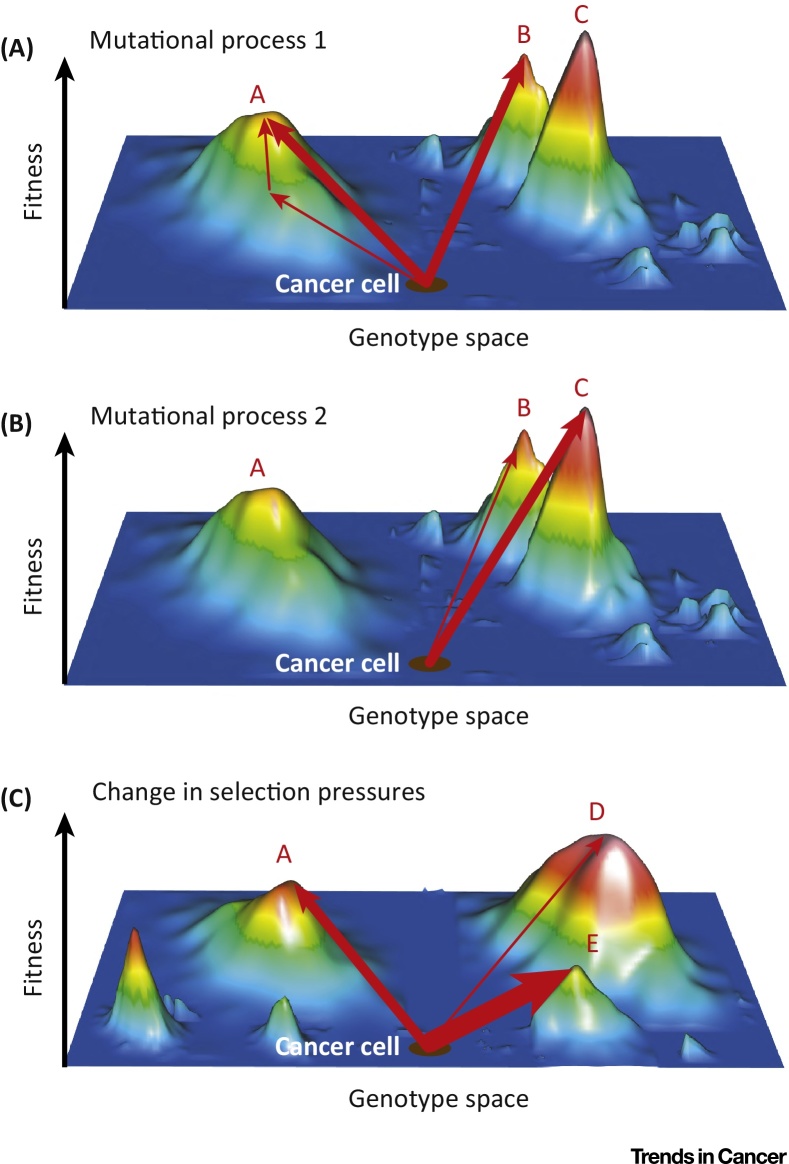
Shape and Accessibility of Peaks on a Fitness Landscape Influence the Probability of Distinct Evolutionary Outcomes. The fitness of all possible genotypes in a cancer is displayed on the vertical axis. Highly fit genotypes appear as peaks on this hypothetical fitness landscape. Red arrows indicate the movement on the fitness landscape through the acquisition of a single mutation. (A) Peaks A and B are equally likely to be accessed through a single new mutation (thick arrows). Peak A can alternatively be accessed by a combination of two mutations that is less likely to occur (thin arrows). (B) Changes of the mutational processes operating in a cancer 19, 26 can alter the accessibility of the fitness peaks but not the topology of the fitness landscape. Peak C can now be accessed, whereas mutations required to climb peak A can no longer be generated. (C) A change in selection pressures changes the topology of the fitness landscape. Mutations allowing access to peak E are now the most likely to occur but they have a lower fitness increment than peaks A or D.

The relative fitness of genetic alterations can be illustrated as a multidimensional fitness landscape, which is a simplified graphic representation of fitness as a function of genotype [94] (Figure 5). The topology of the fitness landscape influences the probability that evolution takes a specific path among competing possibilities. For example, if two advantageous mutations are equally likely to be generated by the operating mutational processes, the one that displays a higher fitness increment on the fitness landscape is less likely to be eradicated by drift. Thus, it will evolve more frequently and it will also proliferate and emerge faster.

The ability to observe cancer evolution repeatedly in thousands of patients with a given cancer type provides the opportunity to empirically delineate these fitness landscapes. The systematic interrogation of drug resistance genotypes already started to probe the complexity of these landscapes 45, 95 and identified a limited but nevertheless challenging number of distinct resistance driver genes and mutations. Although phenotypic convergence with pervasive restoration of signaling through a particular resistance pathway has been identified in many cancer types 13, 45, 51, 95, 96, genotype predictability remains poor for individual patients, most likely owing to the influence of stochastic processes.

The number of patient samples that will need to be sequenced to map complex fitness landscapes in tumors with many infrequently occurring drug resistance drivers may not be achievable in practice. Large-scale mutagenesis, RNAi, and CRISPR/CAS screens of cancer cell lines 97, 98, 99, 100, and of more realistic laboratory tumor models, such as patient-derived xenografts [101], or 3D primary cultures [102] are additional powerful tools to achieve this. Reconstructing fitness landscapes for each cancer type and linking them with information about current mutation processes and the cancer cell population size in an individual patient should allow estimation of the probability of distinct fitness solutions and increasing genotype predictability for precision cancer medicine. Epistatic interactions between mutations influenced the availability of adaptive trajectories in microbial evolution experiments [103] and the order in which Janus kinase 2 (*JAK2*) and tet methylcytosine dioxygenase 2 (*TET2*) driver mutations were acquired in myeloproliferative neoplasms altered their biology, clinical features, and future evolutionary paths [104]. Incorporating such epistasis interactions into cancer fitness landscape models will also be crucial.

Prediction approaches based on fitness landscapes could prioritize an individual cancer for pre-emptive targeting with drugs that are effective against the anticipated resistance genotypes. Similar approaches are being pursued for the prediction of antibiotics resistance and evolution in other contexts and many methods are translatable 94, 105, 106.

## Concluding Remarks

The development of a coherent cancer evolutionary framework that is amenable to theoretical and computational modeling is critically important to realize more accurate predictions. This model needs to incorporate the spatial constraints in solid tumors and optimal sampling approaches and parameter sets that need to be measured in a tumor to inform such predictive models need to be defined. Input parameters will most likely be tumor type-specific to take variability in growth, migration, metastasis, and driver landscapes into account. These and other questions need to be addressed to expand the predictability horizon (see Outstanding Questions). However, evolution remains centrally influenced by stochastic effects and exact measurements of the entire clonal composition of a cancer will not be possible in relevant clinical scenarios. These fundamental characteristics will continue to limit predictability in precision cancer medicine.

Outstanding Questions Can we develop realistic cancer evolution models incorporating 3D structures and empirical fitness landscapes to make predictions?

What macroscale and microscale parameters need to be measured to feed these cancer evolution forecasting models?

How does the strength of selection pressures influence cancer evolution trajectories?

Which clinically relevant outcomes such as resistance genotype, time to resistance, probability of recurrence, or metastasis development can be forecasted most accurately?

What are the most sensitive methods to detect subclones growing deterministically?

How can ctDNA-based clonal dynamics analysis and rebiopsies best be combined to reconstruct empirical fitness landscapes of drug resistance evolution?

How do measurement errors of the clonal composition influence predictions? Cancer is a non-linear system as a result of the ability of cancer cells to proliferate exponentially. Small measurement errors of the starting conditions may lead to major deviations in predictions from real outcomes, similar to other systems showing chaotic behavior.

How can we restrain cancer evolvability to prolong time to resistance development and prevent polyclonal resistance?

## Glossary

Deterministic process: a process whose outcome is determined by its initial state.
Stochastic process: a process that can have different outcomes even if the initial state is identical.
